# Comparison of Subcutaneous versus Intramuscular Dexmedetomidine–Midazolam–Ketamine–Morphine (DMKM) Mixture as Chemical Restraint for Endoscopic Sex Determination in Aldabra Giant Tortoises (*Aldabrachelys gigantea*)

**DOI:** 10.3390/ani13233626

**Published:** 2023-11-23

**Authors:** Marco Masi, Alessandro Vetere, Jacopo Casalini, Flavia Corsi, Francesco Di Ianni, Giordano Nardini

**Affiliations:** 1 Centro Veterinario Specialistico, Via Sandro Giovannini 51/53, 00137 Roma, Italy; apophismasi@gmail.com; 2 Department of Veterinary Science, University of Parma, 43126 Parma, Italy; 3 Clinica Veterinaria Modena Sud, Piazza Dei Tintori 1, 41057 Spilamberto, Italy; 4 Institute of Molecular Biotechnology of the Austrian Academy of Sciences (IMBA), Vienna BioCenter (VBC), Dr. Bohr-Gasse 3, 1030 Vienna, Austria

**Keywords:** DMKM, *Aldabrachelys gigantea*, anaesthesia, chelonians, reptiles

## Abstract

**Simple Summary:**

Anaesthesia in chelonians poses a challenge due to their resistance, making injectable anaesthesia highly valuable for this order of reptiles. In this study, we evaluated the effects of a dexmedetomidine–midazolam–ketamine–morphine (DMKM) combination delivered subcutaneously (SC) or intramuscularly (IM) in twenty-one Aldabra giant tortoises (*Aldabrachelys gigantea*) as a chemical restraint for a minimally invasive procedure for celioscopic sex identification. The intramuscular administration of a DMKM combination seems to be a good option to be used as a safe, reliable, short-lasting anaesthesia for minimally invasive procedures such as celioscopy.

**Abstract:**

Sex identification through coelioscopy is a minimally invasive surgical technique used to determine the sex of chelonians by directly visualizing their internal reproductive organs. An adequate anaesthesiologic plan is essential to guarantee patient immobilization and proper analgesia during the entire surgical procedure. In this study, we evaluated the effects of a combination of dexmedetomidine (0.05 mg/kg), midazolam (1 mg/kg), ketamine (8 mg/kg), and morphine (1 mg/kg) (DMKM) randomly delivered intramuscularly (IM) or subcutaneously (SC) in twenty-one Aldabra giant tortoise (*Aldabrachelys gigantea*) into the right antebrachium for celioscopic sex identification. Heart rate (HR), respiratory rate (RR), and body temperature (BT) were measured, along with the skeletal muscle tone of the thoracic and pelvic limbs, neck retraction reflex, palpebral reflex, and jaw tone every 15 min. The anaesthesiologic plan was considered to be adequate at the loss of the thoracic and pelvic limb retraction reflexes. After a 45 min interval, if the anaesthetic plan was deemed insufficient for the celioscopic procedure, a 5 mg/kg dose of propofol was administered intravenously into the subcarapacial venous plexus. At the end of the procedure, atipamezole (0.5 mg/kg) and flumazenil (0.05 mg/kg) were administered intramuscularly into the left antebrachium as reversal agents. Both HR and RR decreased from baseline to both 15 and 30 min. Due to the persistence of thoracic and pelvic limb retraction reflexes 45 min after DMKM administration, 6/11 (55%) cases in the SC group required the additional administration of propofol, in contrast to only 1/10 (10%) cases in the IM group (*p* = 0.05). The recovery times were comparable between the successfully induced animals in the IM and SC groups. In this study, the intramuscular administration of a DMKM combination quickly produced chemical restraint, suitable for celioscopic sex determination.

## 1. Introduction

*Aldabrachelys gigantea*, commonly known as the Aldabra giant tortoise, is a critically endangered species of tortoise native to the Aldabra Atoll in the Seychelles [1,2,3]. Currently, ongoing conservation measures are crucial to ensure the long-term survival of this species [4,5]. Securing the preservation of these tortoise species heavily depends on the implementation of effective captive breeding programs. Therefore, it is imperative for these programs to adhere to optimal management practices, given the significance of population density and the sex ratio of the specimens [5,6,7]. Immature Aldabra giant tortoises are not sexually dimorphic, reaching sexual maturity between the ages of 20 and 25 years, although this can sometimes take longer [8]. In clinical practice, the sex of immature chelonians can be easily identified endoscopically [9]. Celioscopic sexing is a minivasive technique that involves making a small incision in the body wall of the anaesthetized chelonian in the prefemoral fossa for coelomic access. A rigid endoscope is then inserted and used to directly identify the gonads and reproductive structures for accurate sex identification [9]. Administering anaesthesia for coelioscopy in chelonians is a critical aspect to ensure the safety and well-being of the animal during the procedure. Anaesthesia allows the chelonian to be relaxed and unconscious, preventing pain and discomfort during the coelioscopic examination [9,10,11,12]. Injectable anaesthetics are commonly used for chelonians undergoing coelioscopy [11]. Some commonly used anaesthetic agents for chelonians include injectable opioids, dissociative anaesthetics, and injectable or inhalant induction agents [10,12]. These medications are typically administered intramuscularly or intravenously to induce and maintain anaesthesia [10]. Because reptiles have a notoriously prolonged recovery, it is important to choose a suitable combination of drugs that should have wide safety margins, should be short-acting, and should be reversible where possible, promoting more rapid recovery after anaesthesia [10,12,13,14]. Dexmedetomidine, an a2-adrenergic agonist, provides sedation, muscle relaxation properties, and analgesia in many species, reptiles included [10]. Dexmedetomidine is frequently used in conjunction with either ketamine or benzodiazepines to minimize the required drug dosage [10,11,12]. Midazolam is a short-acting, water-soluble benzodiazepine with sedative, anticonvulsant, amnestic, anxiolytic, and skeletal muscle relaxant properties, with minimal effects on the cardiopulmonary system [15]. It is efficiently absorbed when administered intramuscularly (IM) or subcutaneously (SC), exhibiting limited impact on cardiovascular and pulmonary functions. Its effects can be reversed with flumazenil [15,16]. When utilized as the sole agent, midazolam frequently offers merely mild or unreliable sedation in reptiles [10]. Its use in combination with opioids and other injectable agents (e.g., ketamine, medetomidine, and dexmedetomidine) in a Galapagos tortoise (*Chelonoidis nigra*) and three African spurred tortoises (*Geochelone sulcata*) has been reported [16,17]. Ketamine is a dissociative anaesthetic and N-methyl-D-aspartate receptor antagonist. In snapping turtles (*Chelydra serpentina*), the coadministration of ketamine and midazolam produced more profound effects than either drug alone [18]. Similarly, in loggerhead sea turtles (*Caretta caretta*), ketamine and medetomidine alone achieved less sedative effects than a combination of the two, highlighting the benefits of multimodal mechanisms of action [19]. Morphine is a mu opioid receptor agonist that offers strong pain-relieving properties in a variety of veterinary species [10,12,20]. Substantial evidence suggests that morphine could prove to be a viable analgesic option for certain reptile species, chelonians included [20,21]. In clinical doses, it is typically associated with minimal respiratory side effects [20]. Nevertheless, in certain species, higher dosages can lead to pronounced respiratory depression [21,22]. The onset of action following morphine administration can be notably extended (2–8 h), and the duration of its effects may exhibit significant variability across different species [21,22]. In a recent study involving 10 hatchling green sea turtles (*Chelonia mydas*), researchers found that the intramuscular administration of ketamine–medetomidine–tramadol was a reliable and safe anaesthesia protocol. The turtles displayed no response to any of the potentially painful stimuli, indicating the effectiveness of the anaesthesia. However, it should be noted that anaesthesia did result in apnoea throughout the procedure [23]. In another case report of a leopard tortoise (*Stigmochelys pardalis*), a subcutaneous coadministration of medetomidine–midazolam–ketamine produced 30 min of deep sedation, which was sufficient to allow endoscopy-guided ectopic egg removal from the urinary bladder [24]. Propofol is an intravenous anaesthetic agent used extensively in reptilian anaesthesia because of its rapid onset of the anaesthetic effect and short recovery times. This expedites endotracheal intubation, which can then be followed by the maintenance of anaesthesia through the use of an inhalation agent [14]. However, rapid administration of propofol can lead to respiratory depression [25]. Propofol requires intravascular or intraosseous administration, which can be challenging in chelonians. Intrathecal propofol injection is reported in chelonians and can lead to various complications, such as fore and limb paresis, coma, and spinal necrosis [10]. The objective of this study was to investigate the hypothesis that combining dexmedetomidine, midazolam, ketamine, and morphine (DMKM) would result in efficient anaesthesia for short and minimally invasive procedures, such as celioscopic sex identification, without requiring additional propofol as an induction agent. This combination was aimed at reducing the dosage of each drug while taking advantage of their multimodal mechanisms of action. Additionally, we evaluated two different routes of administration: subcutaneous (SC) and intramuscular (IM).

## 2. Materials and Methods

### 2.1. Animals

Ethical approval for the study was given by the University of Parma (PROT. N. 04/CESA/2023). The owner gave informed consent to allow participation of their animals in the study.

Twenty-one captive-bred, clinically healthy Aldabra giant tortoises (*Aldabrachelys gigantea*) (AGTs) with a median body weight of 2090 g (IQR: 1390–4480 g; range: 890–7190 g) and aged between three and five years were included in the study. Exclusion criteria included any history or visible signs of systemic illness (e.g., signs of infection, external wounds, inflammation, trauma, or neoplasia) or discomfort. No animals were excluded in this study. All animals were divided individually into different plastic enclosures and identified with progressive identification numbers. The animals were kept under controlled environmental conditions, with a room temperature of 27 °C and a 12 h:12 h light/dark cycle [26], and housed in a plastic tank of 3 × 2 × 1 m. They were fed a diet consisting of hay and fresh greens and were given free access to water. Food was withheld 4 days before the procedure to decrease the size of the gastroenteric tract, enhancing endoscopic visibility of the reproductive tract. Every box had a heat bulb as a light source (100 W) and a heat light bulb for basking (31–34 °C). Each animal was weighed, and accurate physical examinations were performed. The animals were soaked daily in a few cm of warm water so that they could rehydrate and defecate. Exclusion criteria included any history of systemic illness or abnormalities, including dyspnoea, evidence of infection, traumatic injuries, and abnormal posture.

### 2.2. Study Design

**Figure 1:**
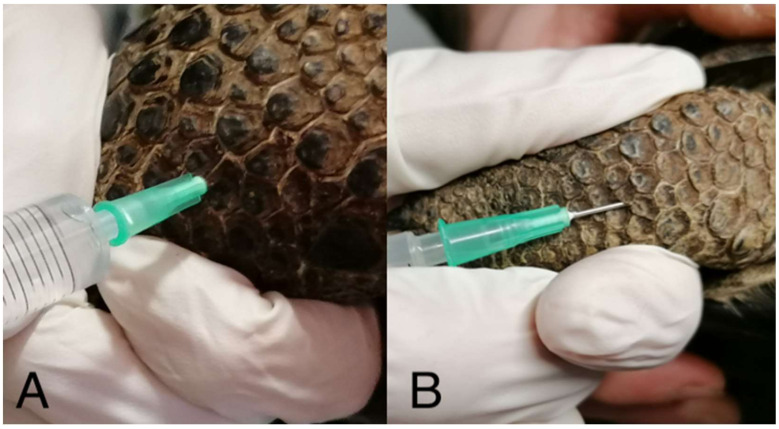
In the IM route (**A**), the needle was inserted for the entire length perpendicularly in the long axis of the right antebrachium, while in the SC route (**B**), the needle was inserted subcutaneously for 2–3 mm parallel to the long axis of the antebrachium.

A mixture of dexmedetomidine (0.05 mg/kg; Dexdomitor 0.5 mg/mL, Vétoquinol Italia S.r.l., Bertinoro, FC, Italy), midazolam (1 mg/kg; Midazolam Hameln ha 5 mg/mL, Hameln Pharmaceuticals Gloucester Business Park, Gloucester, UK), ketamine (8 mg/kg; Lobotor 100 mg/mL, Acme S.r.l., Via Fabrizio De Andrè 8, Milano, Italy), and morphine (1 mg/kg; Morfina Cloridrato Monico 10 mg/mL, Monico s.p.a, Via Ponte di Pietra 7, Venezia, Italy) was delivered to each tortoise in the right antebrachium using a syringe equipped with a 21 gauge × 16 mm needle (IV Needle; Rays spa, Via Francesco Crispi 26, Osimo, Italy) (Figure 1).

**Figure 2:**
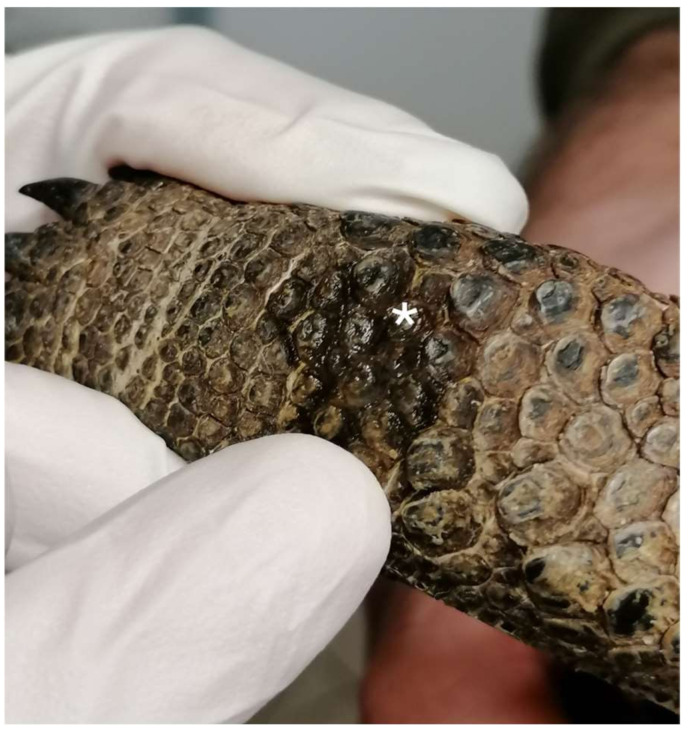
A small amount of drug mixture leaked from the injection site in both the IM and SC groups (white asterisk).

Prior to injections, the skin was scrubbed using 2% chlorhexidine solution (Clorexinal 2%, Nuova Farmec, Settimo di Pescantina, VR, Italy). The route of administration (subcutaneously (SC) or intramuscularly (IM)) was randomly chosen for each tortoise before coelioscopy via simple randomization using randomizer software (www.randomizer.org, accessed on 20 January 2023). Before the injection, aspiration was performed to ensure whether a blood vessel had been accidentally punctured. In the case of accidental blood aspiration, the drug mixture would not be delivered, and the procedure would be repeated. Following drug administration, mild pressure was applied to the injection site using the thumb for a duration of 10 s to minimize the potential for drug leakage. However, unquantifiable drug leakage was noted in both groups of animals (Figure 2).

**Figure 3:**
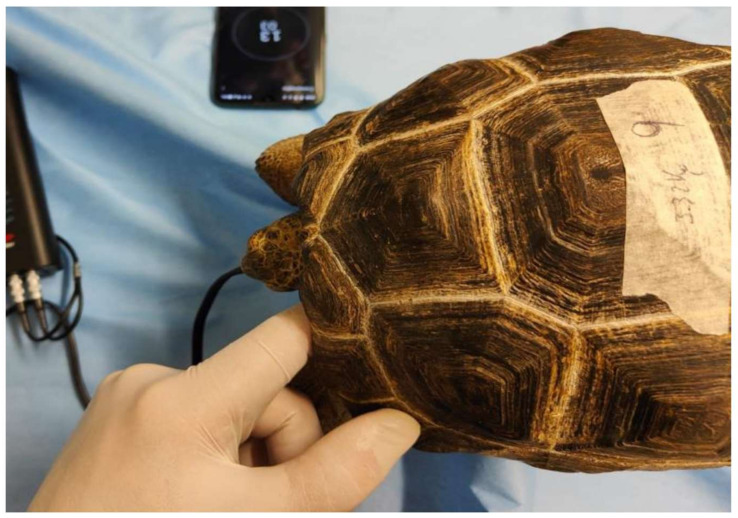
The ultrasonic Doppler probe was positioned in the left cervicobrachial acoustic window.

The time of drug administration was designated as time 0 (T0). The baseline heart rate (HR), respiratory rate (RR), and body temperature (BT) were recorded for 60 s prior to drug administration at T0 and then every 15 min until complete recovery of the animal, for a maximum period of 2 h from initial drug injection. HR was measured using an ultrasonic Doppler flow device (Doppler VET-BP, 8 mhz, Alcyon Italia, Via Nicotera 29, Rome, Italy) directed towards the heart in the cervicobrachial acoustic window (Figure 3).

RR was monitored by observation of skin movement in the region between the neck and shoulder or the prefemoral fossa. BT was monitored using a digital thermometer with an iron external probe (Terracheck, TFA, Herp Italia, Via Giuseppe Verdi 2, Concamarise, Italy) placed inside the cloaca. The following were evaluated every 15 min: palpebral reflex, thoracic and pelvic limb withdrawal reflexes (applying pressure with haemostat forceps), jaw tone, and ease of manual neck extension. Evaluations were performed until complete recovery and for a maximum period of 2 h from drug administration. All reflexes were denoted in a binary way as present (slowed and barely present included) or absent (complete absence of reflexes). The absence of the thoracic and pelvic limb retraction reflexes was considered an adequate anaesthetic plane for the celioscopic procedure. After 45 min, if the anaesthetic plan was considered not adequate for the celioscopic procedure (persistence of the withdrawal reflex or muscle tone of the thoracic and pelvic limbs), a 5 mg/kg propofol dose was administered intravenously in the subcarapacial venous plexus. Tortoises that exhibited apnea (18/21, 85.71%) were intubated with a non-cuffed, silicone endotracheal tube (Foschi s.r.l., Via Livornese est No. 269, Perignano, Italy) of a size appropriate for their glottis diameter and manually ventilated with a bag valve mask (one breath every 30 s) until resumption of spontaneous ventilation. Coelioscopy was performed with the animal placed in right lateral recumbency. The left prefemoral fossa was disinfected with 2.0% chlorhexidine digluconate (Clorexinal 2%, Nuova Farmec, Via Walther Fleming 7, Settimo, VR, Italy). A 0.5 mm skin incision was performed using a scalpel blade No. 11 (Mealli srl, Borgo Santi Apostoli, Firenze, Italy). The transverse and oblique muscles were bluntly dissected pulling the haemostatic forceps in a cranio-dorsal direction, reaching the coelom. A 2.7 mm, 30° viewing rigid endoscope (Storz Telepack TP100 EN, Karl Storz Endoscopia Italia S.r.l., Rome, Italy) housed within a 4.8 mm operative sheath was used to detect and identify the gonads [9]. At the end of the procedure, atipamezole (0.5 mg/kg; Antisedan 5 mg/mL, Vétoquinol Italia S.r.l.) and flumazenil (0.05 mg/kg; Anexate 0.5 mg/5 mL, AVAS Pharmaceuticals S.r.l., Milano, Italy) were administered intramuscularly in the left antebrachium as reversal agents. The recovery time was measured from the time of reversal agent administration. A tortoise was considered to have fully recovered from sedation or anaesthesia when it exhibited all assessed reflexes or responses, when the presence of palpebral reflex, thoracic, and pelvic limb withdrawal reflexes was indicated (either reduced or intact, as opposed to absent), and when the animal could maintain its head above ground and initiate spontaneous movement. During recovery, the tortoises were placed individually in a plastic tank of 3 × 2 × 1 m with a disposable absorbent pad as a substrate. Environmental conditions were the same as before the endoscopic procedure. Thirty-six hours after the end of the procedures, all of the animals ate and were returned to the owner.

**Figure 4:**
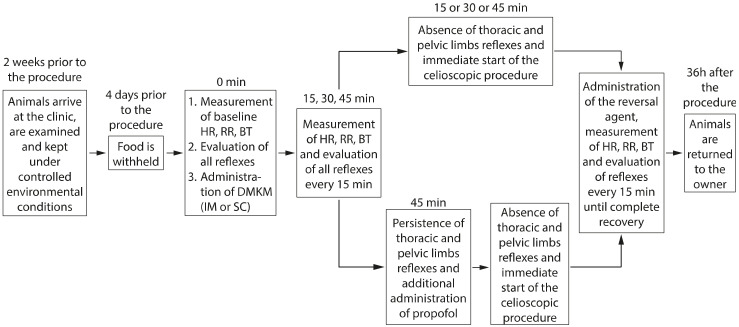
Timeline diagram of the procedure.

A timeline diagram of the whole procedure is represented in Figure 4.

### 2.3. Statistical Analysis

Statistical analysis was performed using R software (version 3.6.3). The data are reported herein as the median, interquartile range (IQR = 25th–75th percentiles), and range. Time to loss and time to recovery of reflexes are reported as the grouped median, grouped interquartile range (IQR = 25th–75th percentiles), and range, to take into account the fact that timepoints were taken at discrete intervals of 15 min, and are compared using the Wilcoxon signed-rank test. Heart rate (HR), respiratory rate (RR), and body temperature (BT) were rank transformed and compared between groups and timepoints using pairwise *t* tests. The route (SC vs. IM) of DMKM administration (independent variable) was evaluated as a predictor for the loss of each reflex and the need for additional propofol administration (dependent variables) in logistic regression models. No threshold on the *p*-value was used to assess statistical significance, since there is increasing evidence that this practice should be avoided. Instead, we still report *p*-values in some cases together with effect sizes (odds ratios (ORs)) and 95% confidence intervals (CIs) which should always be considered all together along with other factors (e.g., related prior evidence, plausibility of mechanism, study design, and data quality, etc.) to interpret the meaningfulness of the findings in a critical manner [27,28,29].

## 3. Results

**Figure 5:**
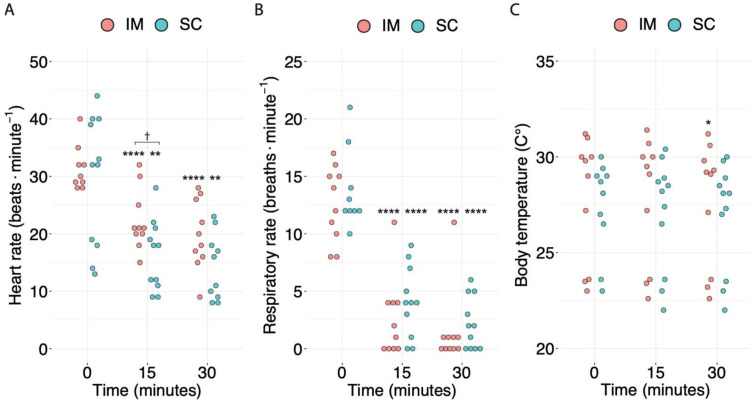
Distribution of the (**A**) heart rate, (**B**) respiratory rate, and (**C**) body temperature over time. For each timepoint, the values for the intramuscular (IM) (*n* = 10) and subcutaneous (SC) (*n* = 11) groups are represented in pink (on the left) and light blue (on the right), respectively. Asterisks (*) indicate the order of magnitude of the *p*-value between the baseline (0 min) and later timepoints within the same treatment. Daggers (†) indicate the order of magnitude of the *p*-value between different routes of administration at the same timepoint. An absence of symbols indicates a *p*-value > 0.05. * *p* < 0.05, ** *p* < 0.01, **** *p* < 0.0001, † 0.01 < *p* < 0.05.

Out of the 11 tortoises, 7 (64%) belonging to the IM group were identified as male, and 4 (36%) tortoises belonging to the SC group were identified as female. The intramuscular (IM) and subcutaneous (SC) groups consisted of 10 and 11 animals, respectively. No difference was observed in body weight (g) between the two groups (SC: median = 1970, IQR = 1330–4310, range = 890–7190; IM: median = 2655, IQR = 1728–4190, range = 1370–5760; *p* = 0.5). HR and RR decreased significantly over time in both groups in a comparable manner before the administration of the antagonist to any animal (Figure 5A,B). No differences were observed between the BTs of the two groups (Figure 5C).

**Figure 6:**
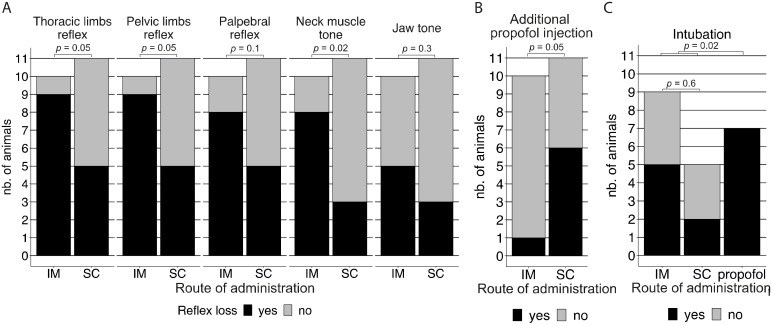
(**A**) Proportion of animals in the intramuscular (IM) (*n* = 10) and subcutaneous (SC) (*n* = 11) groups that lost the thoracic and pelvic limb withdrawal reflexes, palpebral reflex, neck muscle, and jaw tone within 45 min after DMKM administration prior to the decision on additional propofol injection. (**B**) Proportion of animals in the intramuscular (IM) (*n* = 10) and subcutaneous (SC) (*n* = 11) groups that had to be induced with propofol 45 min after the initial DMKM administration. (**C**) Proportion of animals for which intubation was necessary and successful in the intramuscular (IM) (*n* = 9) and subcutaneous (SC) (*n* = 5) groups successfully induced with DMKM after 45 min and the propofol (*n* = 7) group.

In the IM group, 9/10 (90%) animals lost the thoracic and pelvic limb withdrawal reflexes within 45 min after DMKM administration, in contrast to only 5/11 (45%) animals in the SC group (Figure 6A). Hence, due to the persistence of thoracic and pelvic limb withdrawal reflexes 45 min after DMKM administration, the remaining 6/11 (55%) animals in the SC group required the additional administration of propofol to allow the start of the celioscopic procedure after 45 min, in contrast to only 1/10 (10%) animals in the IM group (Figure 6B). These differences between the two groups suggest that, compared to SC administration, the IM administration is 10.8 times (OR = 10.8, 95% CI = 1.33–237.05, *p* = 0.05) more likely to result in loss of the thoracic and pelvic limb reflexes within 45 min from the DMKM administration, allowing one to perform the celioscopic procedure in this timeframe without the additional need for propofol.

Within 45 min after DMKM administration prior to propofol injection, 8/10 (80%) IM and 5/11 (45%) SC cases also lost the palpebral reflex, 8/10 (80%) IM and 3/11 (27%) SC cases lost neck muscle tone, and 5/10 (50%) IM and 3/11 (27%) SC cases lost jaw tone (Figure 6A). For 9/10 (90%) of the IM and 5/11 (45%) of the SC cases for which DMKM was effective within 45 min and did not need to be induced with propofol, there were no remarkable differences in the time to loss of reflexes (Table 1).

In 1/1 (100%) of the IM and 5/6 (83%) of the SC cases successively induced with propofol, all of the measured reflexes were lost within 15 min, allowing the start of the procedure after 4 min from the time of propofol administration for the only IM case and between 4 and 14 min from the time of propofol administration for the 5/6 SC cases. In the remaining 1/6 (17%) SC cases successively induced with propofol, a delayed loss of all reflexes was observed, except for pelvic limb retraction, which was abolished within 15 min, resulting in the procedure being started 24 min after propofol administration.

Among the animals that had to be induced with propofol, 7/7 (100%) also showed apnoea, and the absence of jaw tone allowed successful intubation in all of them (Figure 6C). Among the animals successfully induced with DMKM, 12/14 (86%) of them (8 IM, 4 SC) showed apnoea, but in contrast to the group induced with propofol, only 8/14 (57%) of them (5 IM, 3 SC) lost jaw tone (Figure 6A), and intubation was successful in 7/14 (50%) (5 IM, 2 SC) of the cases (Figure 6C).

No remarkable differences were observed in the average recovery time of the individual reflexes between the IM and SC groups (Table 2). All animals reached a complete recovery of all reflexes within 60 min. Twelve hours after the coelioscopy, all of the animals were active and did not show any abnormal behaviour. No procedure-related complications were noted.

## 4. Discussion

The following databases (Google Scholar, PubMed) were searched with the following keywords: “DMKM, anaesthesia, *Aldabrachelys gigantea*, SC, IM, dexmedetomidine, ketamine, midazolam, morphine”; one textbook was consulted [30]. No studies about DMKM as an anaesthetic protocol in this species were found in these searches. In this study, we administered a DMKM combination IM or SC in young and not sexually dimorphic Aldabra giant tortoises. In a study by Lahner et al. [31], the induction times following subcutaneous administration of ketamine–dexmedetomidine to red-eared slider turtles (*Trachemys scripta elegans*) were notably longer and exhibited greater variability than those following intramuscular injection. Instead, the level of anaesthesia attained within the initial 45 min displayed no distinction between intramuscular and subcutaneous injection [32]. In another study on leopard geckos, the administration of alfaxalone–midazolam via SC injection resulted in the first effects occurring within 5 to 10 min [33]. A comparison between routes of administration of drugs in reptiles has not been well studied, and this is typically dependent on species and related to anatomical variability [33]. Historically, IM administration has been the favoured method for delivering anaesthetic and analgesic drugs to reptiles. SC administration has generally been disregarded due to the belief that its lower vascularity might result in extended and less consistent onset times, metabolism, and elimination when contrasted with the IM route [34]. The contemporary literature persists in discussing the potential for variable absorption with SC drug administration, even though current research demonstrates that anaesthetic and analgesic effects achieved through SC drug administration in reptiles are both prompt and consistent. Moreover, no noteworthy disparity in anaesthetic depth has been observed when contrasting SC and IM induction methods [10,32,33]. Additionally, in reptiles, the advantages associated with SC administration, when contrasted with IM administration, encompass the consistent accessibility of subcutaneous space across different species, the capacity to administer large volumes, and the opportunity to utilize a range of subcutaneous sites [10]. However, in this study, the subcutaneous space in the antebrachium was chosen as the injection site of the SC group instead of the region set in the front half of the body, between the neck and the forelimb, where the cutis is more distensible. This choice was made because clinically healthy tortoises tend to strongly retract their limbs into the shell, exposing only a small portion of the cutis of the dorsal surface of the forelimb. Every attempt to force the forelimbs out would have been either traumatic for the animal or unsuccessful. In every animal belonging to both the IM and SC groups, a small, unquantifiable amount of DMKM leaked from the injection site. This was supposedly related to the poor distensible skin and the well-developed antebrachial muscles, which are typical features of large terrestrial chelonians [34], in relation to the volume of the injected drug mixture. In a recent study evaluating the effect of the IM dexmedetomidine–midazolam–ketamine (DMK) combination in red-footed tortoises, the median time to maximum drug effect was reached in approximately 35 min [35]. In the literature, as there are no available studies about the effects of DMKM in Aldabra giant tortoises, a limit of 45 min was chosen as a cut-off for the time to loss of reflexes, and in the case of persistent thoracic and pelvic limb withdrawal reflexes, the animal would be induced with IV propofol. In this study, we found that the number of animals for which the anaesthesiologic plan was adequate for the celioscopic procedure within 45 min of DMKM administration was notably higher in the IM (9/10 (90%) cases) group than in the SC group (5/11 (45%) cases). For the subset of cases successfully induced with DMKM either SC or IM, the time to loss of reflexes was comparable between the two groups of animals. The signs used to assess the level of sedation in reptiles are fairly consistent. The loss of withdrawal reflexes and muscle tone is expected to follow the following sequence: pectoral limbs, pelvic limbs and neck, tail and cloaca, and finally jaw tone [36]. In this case, only 8/14 (57%) animals (5 IM, 3 SC) that were successfully induced through IM and SC DMKM lost jaw tone, and in only 7/14 (50%) (5 IM, 2 SC) of these animals, intubation was successful. During induction, the jaw reflex is often lost last, indicating a deeper state of narcosis and allowing intubation and manual ventilation [37]. Additionally, among the animals successfully induced with IM DMKM, one tortoise did not lose the neck retraction reflex, and another did not lose the palpebral reflex, indicating a lighter plane of anaesthesia. HR and RR decreased significantly over time in both groups before the start of the celioscopic procedure, as also reported in the study by Eshar et al. [35] using IM DMK administration in captive red-footed tortoises (*Chelodinis carbonaria*). In dogs and cats, the pharmacokinetics of anaesthetic drugs are influenced by the route of administration [35]. Specifically, the subcutaneous route does not always allow for complete and rapid uptake of the drug mixture. Interaction with the α-2B receptors of the precapillary sphincter in peripheral vascular beds by α-2 receptor agonists could lead to a reduction in peripheral drug absorption [36,37,38]. This mechanism may have contributed to the reduction in systemic absorption of MKM when mixed with dexmedetomidine in the subcutaneous group, with a consequently less sedative effect.

Propofol is commonly used alone as an induction agent in chelonians at 10 to 20 mg/kg [10] or combined with other injectable agents at a lower dosage [39,40]. A dosage of 5 mg/kg was chosen as a DMKM mixture that was previously administered, avoiding possible complications such as prolonged recovery after the coelioscopy or a dose-dependent depressant respiratory function [41,42]. In fact, all of the animals that needed propofol also lost jaw tone and were intubated, compared to 8/14 (5 IM 3 SC) tortoises in the IM and SC groups which lost jaw tone, allowing 7 of them (5 IM, 2 SC) to be intubated. Apnoea occurs in reptiles in the surgical and deep plane of anaesthesia; if they are breathing spontaneously, the anaesthetic plane should be considered to be light [10]. However, even though measuring pain and analgesia is challenging in reptiles [42], as no movements or changes in the HR were noted during the surgical procedures, the analgesia was considered adequate. Moreover, no change in the normal behaviour was noted 12 h postoperatively and 36 h after the coelioscopy, and all of the animals ate, displaying no visible signs of discomfort or lameness [42]. No swelling in the injection site was noted in any of the animals at 12 and 36 h after the coelioscopic procedure. Coelioscopy is considered a minimally invasive surgical procedure [9]. However, multimodal anaesthesia was chosen to provide a good analgesic level throughout the entire surgical procedure. In fact, a DMK combination provided sufficient sedation or light anaesthesia, allowing the execution of various clinical procedures, including minimally invasive endoscopic examinations, blood sample collection, and imaging [18,24,27,43,44]. The authors of prior studies focusing on the anaesthesia of red-footed tortoises used higher doses of dexmedetomidine–midazolam and ketamine than those used in the present DMKM protocol [35,44] and did not include morphine in the drug mixture. In this case, morphine was chosen as an additional pain relief agent, as this has been demonstrated to be effective in several reptilian species [18]. Both opioids and a2-adrenoceptor agonists can induce respiratory depression in reptiles [10,12,45,46,47]. The occurrence of apnoea after the administration of medetomidine and dexmedetomidine is reported in chelonians [13,35,46]. In this study, 12/14 tortoises belonging to the IM and SC groups (8 IM, 4 SC) had apnoea and needed intubation and manual ventilation. The additional use of morphine in the DMK mixture should cause more respiratory depression than using a2-adrenoceptor agonists alone. For this reason, breathing assessment should always be performed and the patient should be intubated and ventilated when needed. However, the recovery was smooth and rapid after the reversal administration, and no other apparent adverse effects were noted. This investigation was a clinical study performed during sex identification in 21 giant Aldabra tortoises. To detect possible complications, follow-up studies including several individuals are needed. Moreover, an experimental setting should be designed to evaluate the cardiocirculatory and analgesic effects of DMKM mixture in terms of its pharmacokinetic and pharmacodynamic properties in this species.

## 5. Conclusions

In this study, a DMKM combination delivered IM resulted in deep sedation or light anaesthesia lasting for the duration of the celioscopic procedure and a smooth and rapid recovery after reversal administration. As more than half of the animals belonging to the SC group needed an additional intravenous administration of propofol to reach the surgical plane, this route of administration should be discouraged.

## Figures and Tables

**Table 1:** Time to loss of reflexes only including subjects successfully induced with DMKM within 45 min (9 IM, 5 SC). The median (IQR = 25th–75th percentiles) was calculated only including the subset of animals within the ones successfully induced with DMKM which lost the stated reflex within 45 min after DMKM administration.

Parameter	IM DMKM	SC DMKM	*p*-Value
Time to loss of the thoracic limb reflex for positive cases (minutes)	16.9 (8.4–25.3) (*n* = 9/9)	12.5 (6.3–20.6) (*n* = 5/5)	0.6
Time to loss of the pelvic limb reflex for positive cases (minutes)	16.9 (8.4–25.3) (*n* = 9/9)	17.5 (9.4–23.8) (*n* = 5/5)	1
Time to loss of the palpebral reflex for positive cases (minutes)	18.8 (10–26.25) (*n* = 8/9)	18.8 (9.4–28.1) (*n* = 5/5)	1
Time to loss of neck muscle tone for positive cases (minutes)	30 (15.0–37.5) (*n* = 8/9)	7.5 (3.8–21.2) (*n* = 3/5)	0.06
Time to loss of jaw tone for positive cases (minutes)	22.5 (9.4–36.6) (*n* = 5/9)	18.8 (11.3–24.4) (*n* = 3/5)	0.8

**Table 2:** Time to resumption of reflexes after loss. Medians (IQR = 25th–75th percentiles) were calculated only including the subset of animals that were successfully induced with DMKM and lost the respective reflexes until the end of the procedure, discarding those that had already started to partially recover the respective reflexes during the administration of the antagonist.

Parameter	IM DMKM	SC DMKM	*p*-Value
Time to resumption of the thoracic limb reflex (minutes)	8.8 (4.4–13.1) (*n* = 7)	22.5 (11.3–33.8) (*n* = 3)	0.2
Time to resumption of the pelvic limb reflex (minutes)	7.5 (3.8–11.3) (*n* = 8)	11.25 (5.6–18.8) (*n* = 3)	0.2
Time to resumption of the palpebral reflex (minutes)	10.5 (5.3–16.9) (*n* =7)	9.4 (4.7–14.1) (*n* = 5)	0.7
Time to resumption of neck muscle tone (minutes)	10.5 (5.3–16.9) (*n* = 7)	11.3 (5.6–18.8) (*n* = 3)	0.9
Time to resumption of jaw tone (minutes)	9.4 (4.7–14.1) (*n* = 5)	7.5 (3.8–11.6) (*n* = 2)	0.8
Time to complete resumption of all reflexes (minutes)	13.1 (6.6–24.4) (*n* = 8)	12.5 (6.3–26.3) (*n* = 5)	1

## Data Availability

Data is contained within the article.

## References

[1] Bour R. (1994). Recherches Sur Des Animaux Doublement Disparus: Les Tortues Gé Antes Subfossiles De Madagascar.

[2] Bunbury N., von Brandis R., Currie J.C., van de Crommenacker J., Accouche W., Birch D., Chong-Seng L., Doak N., Haupt P., Haverson P. (2018). Late stage dynamics of a successful feral goat eradication from the UNESCO world heritage site of aldabra atoll, seychelles. Biol. Invasions.

[3] Gerlach J., Rocamora G., Gane J., Jolliffe K., Vanherck L. (2013). Giant tortoise distribution and abundance in the seychelles islands: Past, present, and future. Chelonian Conserv. Biol..

[4] Gerlach J. (2004). Giant Tortoises of the Indian Ocean: The Genus Dipsochelys Inhabiting the Seychelles Islands and the Extinct Giants of Madagascar and the Mascarenes.

[5] Griffiths O., Andre A., Meunier A. (2013). Tortoise breeding and ‘re-wilding’on Rodrigues Island. Chelonian Res. Monogr..

[6] Gerlach J. (2011). Development of distinct morphotypes in captive seychelles–aldabra giant tortoises. Chelonian Conserv. Biol..

[7] Kuchling G., Griffiths O. (2012). Endoscopic imaging of gonads, sex ratios, and occurrence of intersexes in juvenile captive-bred aldabra giant tortoises. Chelonian Conserv. Biol..

[8] Swingland I.R. (1977). Reproductive effort and life history strategy of the aldabran giant tortoise. Nature.

[9] Hernandez-Divers S.J., Stahl S.J., Farrell R. (2009). An endoscopic method for identifying sex of hatchling Chinese box turtles and comparison of general versus local anesthesia for coelioscopy. J. Am. Vet. Med. Assoc..

[10] Sladky K.K., Mans C. (2012). Clinical anesthesia in reptiles. J. Exot. Pet Med..

[11] Innis C.J., Hernandez-Divers S., Martinez-Jimenez D. (2007). Coelioscopic-assisted prefemoral oophorectomy in chelonians. J. Am. Vet. Med. Assoc..

[12] Read M.R. (2004). Evaluation of the use of anesthesia and analgesia in reptiles. J. Am. Vet. Med. Assoc..

[13] Sleeman J.M., Gaynor J. (2000). Sedative and cardiopulmonary effects of medetomidine and reversal with atipamezole in desert tortoises (Gopherus agassizii). J. Zoo Wildl. Med..

[14] Vigani A. (2014). Chelonia (tortoises, turtles, and terrapins). Zoo Anim. Wildl. Immobil. Anesth..

[15] Dundee J.W., Halliday N.J., Harper K.W., Brogden R.N. (1984). Midazolam. A review of its pharmacological properties and therapeutic use. Drugs.

[16] Aitken-Palmer C., Heard D., Jacobson E., Hall N., Thieman K., Ellison G. (2010). Clinical Management of Cloacal Prolapse in an Adult Galapagos Tortoise (Geochelone nigra).

[17] Mans C., Sladky K.K. (2012). Endoscopically guided removal of cloacal calculi in three African spurred tortoises (Geochelone sulcata). J. Am. Vet. Med. Assoc..

[18] Bienzle D., Boyd C.J. (1992). Sedative effects of ketamine and midazolam in snapping turtles (Chelydra serpentina). J. Zoo Wildl. Med..

[19] Chittick E.J., Stamper M.A., Beasley J.F., Lewbart G.A., Horne W.A. (2002). Medetomidine, ketamine, and sevoflurane for anesthesia of injured loggerhead sea turtles: 13 cases (1996–2000). J. Am. Vet. Med. Assoc..

[20] Sladky K.K., Kinney M.E., Johnson S.M. (2008). Analgesic efficacy of butorphanol and morphine in bearded dragons and corn snakes. JAMA.

[21] Sladky K.K., Miletic V., Paul-Murphy J., Kinney M.E., Dallwig R.K., Johnson S.M. (2007). Analgesic efficacy and respiratory effects of butorphanol and morphine in turtles. J. Am. Vet. Med. Assoc..

[22] Mosley C. (2011). Pain and nociception in reptiles. Vet. Clin. Exot. Anim. Pract..

[23] Scheelings T.F., Gatto C., Reina R.D. (2020). Anaesthesia of hatchling green sea turtles (Chelonia mydas) with intramuscular ketamine-medetomidine-tramadol. Aust. Vet. J..

[24] Mans C., Foster J.D. (2014). Endoscopy-guided ectopic egg removal from the urinary bladder in a leopard tortoise (Stigmochelys pardalis). Can. Vet. J..

[25] Schumacher J., Yelen T. (2006). Anesthesia and Analgesia.

[26] Falcón W., Baxter R.P., Furrer S., Bauert M., Hatt J.M., Schaepman-Strub G., Ozgul A., Bunbury N., Clauss M., Hansen D.M. (2018). Patterns of activity and body temperature of Aldabra giant tortoises in relation to environmental temperature. Ecol. Evol..

[27] White N.M., Balasubramaniam T., Nayak R., Barnett A.G. (2022). An observational analysis of the trope “A p-value of < 0.05 was considered statistically significant” and other cut-and-paste statistical methods. PLoS ONE.

[28] Mc Shane B.B., Gal D., Gelman A., Robert C., Tackett J.L. (2019). Abandon statistical significance. Am. Stat..

[29] Hurlbert S.H., Levine R.A., Utts J. (2019). Coup de grâce for a tough old bull: “Statistically significant” expires. Am. Stat..

[30] Divers S.J., Stahl S.J. (2018). Mader’s Reptile and Amphibian Medicine and Surgery.

[31] Lahner L., Mans C., Sladky K. Comparison of Anesthetic Induction and Recovery Times After Intramuscular, Subcutaneous or Intranasal Dexmedetomidine-Ketamine Administration in Red-Eared Slider Turtles (Trachemys Scripta Elegans). Proceedings of the Conference American Association of Zoo Veterinarians.

[32] Doss G.A., Fink D.M., Sladky K.K., Mans C. (2017). Comparison of subcutaneous dexmedetomidine-midazolam versus alfaxalone-midazolam sedation in leopard geckos (Eublepharis macularius). Vet. Anaesth. Analg..

[33] Hawkins S.J., Cox S., Yaw T.J., Sladky K. (2019). Pharmacokinetics of subcutaneously administered hydromorphone in bearded dragons (Pogona vitticeps) and red-eared slider turtles (Trachemys scripta elegans). Vet. Anaesth. Analg..

[34] Abdala V., Manzano A.S., Herrel A. (2008). The distal forelimb musculature in aquatic and terrestrial turtles: Phylogeny or environmental constraints?. J. Anat..

[35] Eshar D., Rooney T.A., Gardhouse S., Beaufrère H. (2021). Evaluation of the effects of a dexmedetomidine-midazolam-ketamine combination administered intramuscularly to captive red-footed tortoises (Chelonoidis carbonaria). Am. J. Vet. Res..

[36] Berry S.H. (2015). Injectable anesthetics. Veterinary Anesthesia and Analgesia: The Fifth Edition of Lumb and Jones.

[37] Murrell J.C., Hellebrekers L.J. (2005). Medetomidine and dexme-detomidine: A review of cardiovascular effects and antinociceptive properties in the dog. Vet. Anaesth. Analg..

[38] Kallio-Kujala I.J., Raekallio M.R., Honkavaara J., Bennett R.C., Turunen H., Scheinin M., Vainio O. (2018). Peripheral alpha-2-ad- renoreceptor antagonism affects the absorption of intramuscularly coadministered drugs. Vet. Anaesth. Analg..

[39] Porters N., De Rooster H., Bosmans T., Baert K., Cherlet M., Croubels S., Polis I. (2014). Pharmacokinetics of oral transmucosal and in-tramuscular dexmedetomidine combined with buprenorphine in cats. J. Vet. Pharmacol. Ther..

[40] Schumacher J. (2007). Chelonians (turtles, tortoises, and terrapins). Zoo Animal and Wildlife Immobilization and Anaesthesia.

[41] Girling S.J., Raiti P. (2019). BSAVA Manual of Reptiles.

[42] Rodney W., Molly S., Divers S.J., Stahl S.J. (2018). Section 6 Anesthesia. Chapter 48 Sedation. Mader’s Reptile and Amphibian Medicine and Surgery.

[43] Divers S.J. (2015). Endoscopic sex identification in chelonians and birds (psittacines, passerines, and raptors). Vet. Clin. N. Am. Exot. Anim. Pract..

[44] Meireles Y.S., Shinike F.S., Matte D.R., Morgado T.O., Kempe G.V., Corrêa S.H.R., Souza R.L.d., Néspoli P.B. (2016). Ultrasound characterization of the coelomic cavity organs of the red-footed tortoise (Chelonoidis carbonaria). Ciênc. Rural.

[45] Scarabelli S., Di Girolamo N. (2022). Chelonian sedation and anesthesia. Vet. Clin. Exot. Anim. Pract..

[46] Hansen L.L., Bertelsen M.F. (2013). Assessment of the effects of intramuscular administration of alfaxalone with and without medetomidine in Horsfield’s tortoises (Agrionemys horsfieldii). Vet. Anaesth. Analg..

[47] Karklus A.A., Sladky K.K., Johnson S.M. (2021). Respiratory and antinociceptive effects of dexmedetomidine and doxapram in ball pythons (Python regius). Am. J. Vet. Res..

